# Seroprevalence of Human Fasciolosis in Lorestan Province, Western Iran, in 2015–16

**Published:** 2017

**Authors:** Peyman HEYDARIAN, Keyhan ASHRAFI, Mehdi MOHEBALI, Eshrat Beigom KIA, Mojgan ARYAEIPOUR, Ali CHEGENI SHARAFI, Hamid MOKHAYERI, Arezoo BOZORGOMID, Mohammad Bagher ROKNI

**Affiliations:** 1. Dept. of Medical Parasitology and Mycology, School of Public Health, Tehran University of Medical Sciences, Tehran, Iran; 2. Dept. of Medical Parasitology and Mycology, School of Medicine, Guilan University of Medical Sciences, Rasht, Iran; 3. Center for Research of Endemic Parasites of Iran (CREPI), Tehran University of Medical Sciences, Tehran, Iran; 4. Dept. of Communicable Disease Control and Prevention, Deputy of Health, Lorestan University of Medical Sciences, Khorramabad, Iran

**Keywords:** Fasciolosis, Seroepidemiology, Freshwater plants, Iran

## Abstract

**Background:**

The aim of this study was the seroepidemiological survey for detecting the status of human fasciolosis in Lorestan Province, western Iran.

**Methods:**

This cross-sectional study was conducted in 2015–16. Based on statistical estimations, 1256 serum samples were collected from different parts of Lorestan Province, western Iran, and stored at −20 °C until use. The collected serum samples were analyzed at Tehran University of Medical Sciences, Tehran, Iran using indirect ELISA method.

**Results:**

Anti-*Fasciola* antibodies were detected in 16 (1.3%) individuals. Regarding the seropositivity to fasciolosis, no significant differences were found between age groups, sex, level of education and occupation; however significant differences were observed regarding location, consuming local freshwater plants and water resources (*P*<0.02.)

**Conclusion:**

Local freshwater plants and unfiltered water resources were probably the main sources of the infection. Health education by local health centers to elevate awareness of people, and providing facilities for safer drinking water, especially in rural areas may help decrease the risk of fasciolosis infection in this region.

## Introduction

One of the leading zoonotic helminthic diseases is fasciolosis caused by *Fasciola hepatica* and *F. gigantica*. It is listed by WHO among the neglected tropical diseases (1). Furthermore, 17 contaminated million individuals are estimated to be infected and around 180 million individuals living in endemic regions are assumed to be at risk of the diseases (2–4). Human and other mammalian are considered as definitive hosts for fasciolosis infected by eating aquatic plants or by drinking water contaminated with metacercariae (5).

Although fasciolosis is generally considered as a notable veterinary problem, human fasciolosis has recently been regarded as a main health issue in numerous countries (6, 7). According to WHO report, Iran has been placed among six countries recognized to have a serious concern with fasciolosis (8). Before 1989, human fasciolosis was pronounced sporadically in Iran (9–12). Fasciolosis has led to two important epidemics in Iran in 1989 and 1999, respectively, which have been the biggest epidemics of fasciolosis in the history (13). In the recent years, new foci of the disease have been observed in Iran such as Kohgyluyeh va Boyerahmad and Kermanshah provinces (14, 15).

Several techniques including serological and parasitological methods are used for the diagnosis of fasciolosis. Parasitological methods (detection of parasite ova in stool or biliary aspirates) have the highest specificity, but some factors such as low rate of parasite eggs, transient infection, acute and obstructive infections reduce the sensitivity of these methods. Serological tests are usually used for recognition of anti-*Fasciola* antibodies in serum samples in acute phase and in ectopic fasciolosis. These methods are appropriate for diagnosis of chronic fasciolosis by identifying specific antigens in stool samples and antibodies in the serum as well (16). Therefore, serological methods such as ELISA are commonly used for the diagnosis of human fasciolosis in Iran (17, 18).

Because of the specific climatic condition of Iran and occurrence of new foci for fasciolosis (14, 15) the neighboring of Lorestan Province with these new foci, as well as the reports by a previous study revealing *Fasciola* parasite cases from Pirabad village of Doroud city in Lorestan (19), we decided to investigate the seroprevalence of human fasciolosis using indirect ELISA throughout the Lorestan Province, Iran.

## Materials and Methods

### Serum collection

**Fig. 1:**
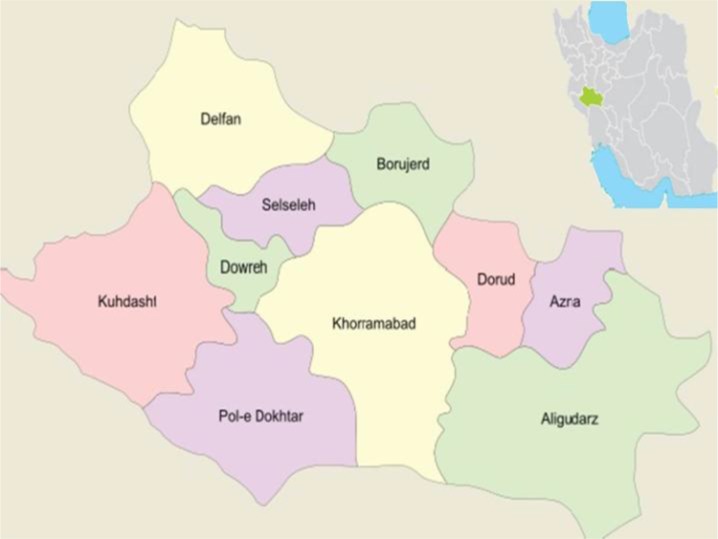
Location of Lorestan Province in Iran

Lorestan Province is located in western Iran (Fig.1). The population of this province, which has ten major cities, is 1,716,527. Overall, 1256 serum samples were collected from the people of Lorestan Province by random stratification recommended by statistician in 2015–16 (Table 1).

**Table 1:** Sample size in different areas of Lorestan Province

	***Aligudarz***	***Borujerd***	***Khorramabad***	***Delfan***	***Dorud***	***Kuhdasht***	***Azna***	***Pol-e-Dokhtar***	***Selseleh***	***Dowreh***
**Urban**	51	140	202	36	60	70	30	43	20	2
**Rural**	29	110	146	54	40	100	20	50	30	23
**Total**	80	250	348	90	100	170	50	93	50	25

The samples were collected in accordance with the population of each city. Based on a random sampling, people were asked to present in health centers for collecting sera. Each patient filled a questionnaire including information on food diet, vegetable consumption, travelling to northern Iran, clinical symptoms etc.

Subjects were informed about the objectives and procedures of the study. They signed a written informed consent. For children this form was taken from parents or their legal guardians. The study was conducted in accordance with the Health Insurance Portability and Accountability Act (HIPAA) guidelines and all procedures were approved by the Ethics Committee of Tehran University of Medical Sciences, Tehran, Iran.

After blood sampling, sera were obtained and stored at −20 °C until use. The collected serum samples were analyzed using ELISA method (17). Finally, absorbance was measured by an ELISA reader at 490 nm.

### Antigen preparation

*Fasciola* infected livers from Tehran slaughterhouses were transferred to the laboratory of Helminthic Diseases, School of Public Health, Tehran University of Medical Sciences, Iran. Parasites were isolated from infected livers and washed for 6 times with normal saline. Afterwards, they were homogenized in 0.045 mM PBS with electrical homogenizer. The supernatant was kept in refrigerator for later usage (17).

### ELISA test

ELISA test was conducted based on previous study with some modifications (17). One hundred microliters of somatic antigen (10 mg/ml) was added to wells of plates and incubated overnight at 37 °C and then 200 microliters of BSA 2% was dispensed to plates. Wells of plates were washed with PBS/Tween 20 for three times. One hundred microliters of a serum samples (diluted1:250) was added to wells coated with antigen and incubated at 37 °C for 30 min. Sera of a *Fasciola*-infected patient and a healthy individual were tested in parallel, as positive and negative controls, respectively. Plates were washed 5 times with PBS/Tween 20. Peroxidase conjugated goat anti-human IgG (diluted 1:10000) was added to wells and incubated at 37 °C for 30 min. After final washing step with PBS/Tween 20, 100 microliters of OPD (o-phenylenediamine dihydrochloride) was added to the wells and reaction was stopped with adding 50 microliters of stopper solution (12.5% H_2_SO). OD was measured at 490 nm with ELISA reader. Cut-off was calculated as X±3 SD.

### Statistical analysis

Statistical analysis was done using SPSS version 22 (Chicago, IL, USA). Chi square test was used for data analyzing. Cut-off was calculated as Mean±3 SD.

## Results

**Fig. 2:**
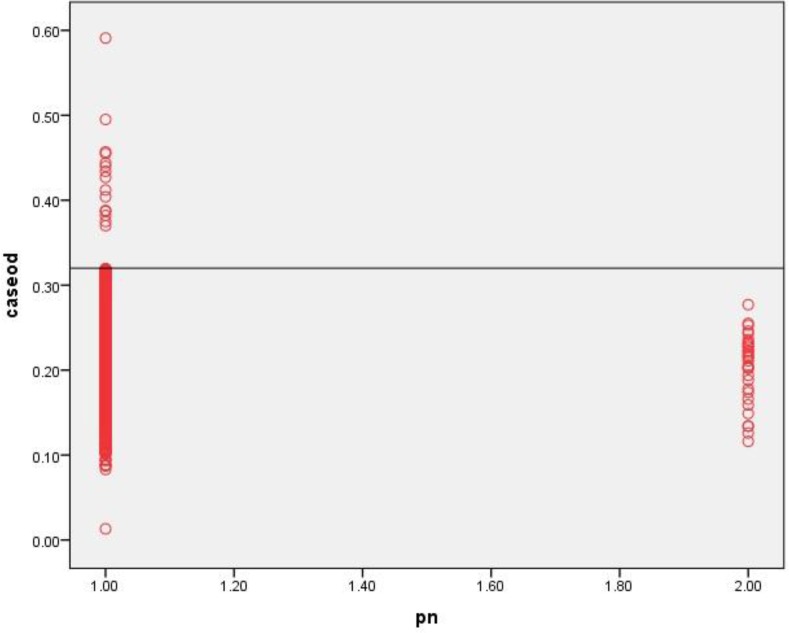
Analysis of sera from subjects and normal controls from, Lorestan Province, Iran using IgG-ELISA. Serum samples gained from subjects (1256 cases, Lanes 1), and normal controls (30, Lanes 2) *pn= patient number

Cut-off for ELISA was calculated as 0.32. Out of 1256 examined cases, 577 (46%) were male and 679 (54%) were female. Overall, 16 persons (1.3%) were serologically positive for fasciolosis. Seropositivity to fasciolosis among the female and male subjects were 1.5% and 1%, respectively, which was not statistically significant (chi square = 0.47, *P*-value = 0.49). Fig. 2 demonstrates the distribution of OD absorbance in subjects and healthy control cases. Table 2 reveals seropositivity rate of fasciolosis in different locations of Lorestan Province and the highest rate was seen in Borujerd district. Table 3 shows the seropositivity in different age groups. The highest rate of seropositivity was seen in ≥ 60 yr old individuals.

**Table 2:** Seropositivity rate of fasciolosis in different locations of Lorestan Province in 2016

***Location***	***Aligudarz***	***Borujerd***	***Khorramabad***	***Delfan***	***Dorud***	***Kuhdasht***	***Azna***	***Pol-e-Dokhtar***	***Selseleh***	***Dowreh***
Seropositivity Rate	0	4%	0	3.3%	2%	0.5%	0	0	0	0

**Table 3:** Seropositivity rate of fasciolosis in different age groups of Lorestan Province in 2016

***Age Groups (yr)***	***Total No.***	***Seropositivity Cases No. (%)***
**≤9**	3	0 (0.0)
**10–19**	85	1(1.2)
**20–29**	398	4 (1)
**30–39**	295	2 (0.7)
**40–49**	189	4 (2.1)
**50–59**	149	2 (1.3)
**≥60**	137	3 (2.2)
**Total**	1256	16 (1.3)

Of sixteen positive cases, seven cases were illiterate, six preliminary educated and three secondary educated. The relation between seropositivity and education level was not statistically significant (*P*-value = 0.08).

Out of sixteen positive cases, ten were housekeepers, one student, two farmers and three self-employed. The relation between seropositivity and occupation was not statistically significant (*P*-value = 0.17).

The prevalence of human fasciolosis in rural and urban areas was 1.9% and 0.4% respectively. Significant relationship between location and fasciolosis infection was observed (chi square = 5.4, *P*-value = 0.02).

The relation between fasciolosis infection and water resource was statistically significant (*P*-value 0.001). The highest prevalence rate of fasciolosis (1.8%), was among people who used spring water. Table 4 shows distribution of positive cases of fasciolosis based on the water resources.

**Table 4:** Distribution of positive cases of fasciolosis based on the water resources

***Water resources***	***Total number***	***Percentage of positive cases***
**Tap water (filtered)**	1106	0.7
**Spring water (unfiltered)**	57	8.1
**Well water (unfiltered)**	47	2.1
**Spring water (filtered)**	19	5.3
**well water (filtered)**	27	3.7
**Total**	1256	1.3

Thirteen of seropositive cases used to eat raw vegetables. Significant relationship was observed between eating raw vegetables and fasciolosis infection (chi square = 18.68, *P*-value < 0.01).

**Fig. 3:**
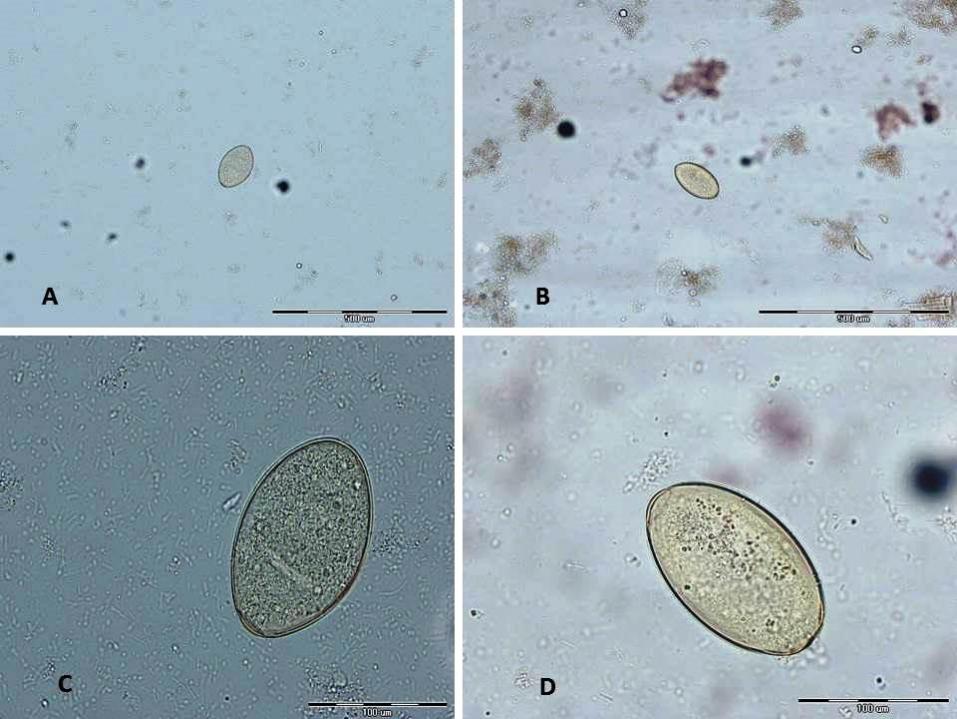
Ova of *Fasciola* in stool examination. A, B: With ×100 magnifications C, D with ×400 magnifications (These pictures were originally captured in this study.)

For all seropositive cases, stool examination was performed 3 times and parasite eggs were observed in four patients (Fig. 3).

Patients diagnosed as positive in this study, were referred to a physician for appropriate treatment. The treatment was a single oral dose of 10 mg of triclabendazole (Egaten) per kilogram of body weight (10 mg/kg). The results were assessed 4 weeks after treatment by stool exam.

## Discussion

In this study, the seroprevalence of human fasciolosis was detected as 1.3% in Lorestan Province, which according to WHO epidemiological classification, is considered “hypoendemic” (6). This study was conducted following the constant studies previously directed in Iran to define the state of human fasciolosis (15, 20). Since six seropositive persons were diagnosed with fasciolosis in one of the villages of Lorestan Province in a previous study (19), the current study was designed to cover the whole province.

While some previous studies have reported the seroprevalence as gender-specific (4, 20–25), no significant difference between genders was observed in our study. This result was in arrangement with several similar studies (13–15, 26–33).

In outbreaks in Kermanshah and Guilan, the highest infection prevalence was observed in 10–19 yr and 10–29 yr respectively (14, 34, 35); while in non-epidemic circumstances, the highest prevalence of infected cases was seen in older age groups (40–59 yr) (28). In this study, highest positive rate was seen in age group of ≥60 yr old. Iran is typically considered as one of the areas where the pattern of fasciolosis infection in adults is more than children (2). This result may be due to non-epidemic causes in this region; older adults are involved in cleaning and washing vegetables for eating and preparing salads and local foods, agricultural activities.

One of the important factors associated with human fasciolosis is dietary habits (36–38). In the current study, statistically significant difference was observed between the prevalence of infection and eating raw vegetables. There are a number of wild aquatic and semi aquatic plants related with human fasciolosis in Iran (13, 15, 37). As noted already, in this province, all seropositive individuals ate *Nasturtium officinalis* (locally name Balmak) which is a popular water plant commonly consumed by local residents. (19). In this area, this plant is considered hypoglycemic which can help treat diabetes or prevent it. In the present study, out of 1256 people, 414 (32.96%) consumed Balmak and among 16 seropositive cases, 13 had a history of eating this vegetable and there was a significant correlation between the eating of Balmak and seropositivity of fasciolosis (chi square = 18.68, *P*-value < 0.01). In the epidemic occurred in 1988 in Guilan Province, 91% of infected individuals had consumed a local plant called ‘’Khalvash” (*Mentha piperita*) (39). However, some studies did not find a significant relationship between consuming raw vegetables and seropositivity of fasciolosis (28, 40, 41).

In this study, statistically significant relationship between water resources and human fasciolosis was observed. The highest rate of the infection was seen in individuals who had used unfiltered spring water (8.1%) and unfiltered well water (2.1%) respectively.

In our study, the highest numbers of infection cases (14 out of 16) were seen in people who lived in rural areas. Fasciolosis mainly is a rural disease and rural people with certain occupations such as farmers and ranchers are at greater risk for infection, because of the closer contact with environmental factors like animal reservoirs, intermediate hosts, the consumption of aquatic plants and drinking unfiltered water (22, 42). Nevertheless Ashrafi et al. found the highest number of human cases in urban areas in Guilan Province and stated that it might be due to the vicinity of rural areas to the cities (28).

This study was the first comprehensive one, which covered all urban and rural areas of Lorestan Province. Limitations of the study might be stated as high costs, which prevented the study team from more sampling.

## Conclusion

Due to high consumption of freshwater plants among seropositive people, it seems that local health centers may play an important role in educating people, especially about the risks of eating raw or uncooked freshwater plants. Local media should also alert people about aquatic plants and encourage public to cultivate freshwater plants in protected areas and fence them off from livestock in order to disrupt the parasite life cycle. Regarding the poor water filtration especially in rural areas and its relation to the prevalence of fasciolosis, it is encouraged that relevant organizations provide facilities for safer water supplies.
